# Different Phenotypes at Onset in Neuromyelitis Optica Spectrum Disorder Patients with Aquaporin-4 Autoimmunity

**DOI:** 10.3389/fneur.2017.00062

**Published:** 2017-02-28

**Authors:** Youming Long, Junyan Liang, Linzhan Wu, Shaopeng Lin, Cong Gao, Xiaohui Chen, Wei Qiu, Yu Yang, Xueping Zheng, Ning Yang, Min Gao, Yaotang Chen, Zhanhang Wang, Quanxi Su

**Affiliations:** ^1^Department of Neurology, the Second Affiliated Hospital of GuangZhou Medical University, Guangzhou, Guangdong Province, China; ^2^Institute of Neuroscience and The Second Affiliated Hospital of Guangzhou Medical University, Key Laboratory of Neurogenetics and Channelopathies of Guangdong Province and the Ministry of Education of China, Collaborative Innovation Center for Neurogenetics and Channelopathies, Guangzhou, China; ^3^Department of Emergency, The Second Affiliated Hospital of GuangZhou Medical University, Guangzhou, Guangdong Province, China; ^4^Department of Neurology, The Third Affiliated Hospital of Sun Yat-Sen University, Guangzhou, Guangdong Province, China; ^5^Department of Neurology, The Affiliated Hospital of Qingdao University, Qingdao, Shandong Province, China; ^6^Department of Neurology, The Fifth Affiliated Hospital of GuangZhou Medical University, Guangzhou, Guangdong Province, China; ^7^Department of Neurology, The Second Chinese Medicine Hospital of Guangdong Province, Guangzhou, Guangdong Province, China; ^8^Department of Neurology, Guangdong 999 Brain Hospital, Guangzhou, Guangdong Province, China; ^9^Department of Neurology, Yunfu City People’s Hospital, Yunfu, Guangdong Province, China

**Keywords:** neuromyelitis optica, aquaporin-4, optic neuritis, myelitis, brain

## Abstract

**Background:**

Although rare, brain abnormalities without optic neuritis (ON) or transverse myelitis (TM) diagnosed with neuromyelitis optica spectrum disorder (NMOSD) have been reported in patients positive for the aquaporin-4 (AQP4) antibody.

**Objective:**

To analyze demographic and clinical differences among NMOSD patients without ON or TM, those with either ON or TM, and patients with simultaneous ON and TM at disease onset.

**Methods:**

In this retrospective study, patients who were positive for the AQP4 antibody, as detected using a cell-based assay, at the Second Affiliated Hospital of Guangzhou Medical University in China were recruited. Demographic and clinical data were obtained from each patient’s medical record.

**Results:**

A total of 292 patients were included in this study and were divided into four subgroups based on their initial manifestations: (i) NMOSD without ON or TM (NMOSD-ON^−^TM^−^, *n* = 70); (ii) NMOSD with ON (NMOSD-ON^+^, *n* = 95); (iii) NMOSD with TM (NMOSD-TM^+^, *n* = 116); and (iv) simultaneous ON and TM [neuromyelitis optica (NMO), *n* = 11]. We found that age at onset was lower in the NMOSD-ON^−^TM^−^ group than that in the other groups. The interval from the first episode to relapse was shorter in the NMOSD-ON^−^TM^−^ group than that in NMOSD-TM^+^ group. Cerebral spinal fluid white cell counts and protein levels were significantly higher in the NMOSD-ON^−^TM^−^ group than those in the other groups. Lower Expanded Disability Status Scale scores were observed in the NMOSD-ON^−^TM^−^ group. Brain abnormalities, including in area postrema and hemisphere lesions, were more frequent in the NMOSD-ON^−^TM^−^ group. Kaplan–Meier analysis showed that patients in the NMOSD-ON^−^TM^−^ group experienced earlier relapse than those in other groups. Conversion to NMO in the NMOSD-ON^+^ group was greater than that in the other groups. Only 14 patients (4.8%, 14/292) had pure brain abnormalities, of which 12 had disease duration of several more years and 8 (57.1%) experienced relapses.

**Conclusion:**

NMOSD patients with different initial manifestations present with significant differences in clinical features during follow-up. Patients with long-term AQP4 autoimmunity in the brain in the absence of ON or TM are not common.

## Introduction

Neuromyelitis optica (NMO) is generally a severe, idiopathic, immune-mediated inflammatory, demyelinating, and necrotizing disease that mainly involves the optic nerve and spinal cord, but rarely the brain. The presence of aquaporin-4 (AQP4) antibody in NMO (1) facilitates its distinction from multiple sclerosis, and many studies have shown that AQP4 autoimmune lesions outside the optic nerve and spinal cord are common (2, 3).

Limited forms of NMO, optic neuritis (ON) or transverse myelitis (TM), positive for the anti-AQP4 antibody are diagnosed as NMO spectrum disorder (NMOSD) (4, 5). Furthermore, increasing numbers of positive cases without optic nerve and spinal cord involvement have been reported, indicating that the requirement for the presence of either ON or TM may confound the definition of NMOSD (6). Previous reports have shown that many manifestations outside the optic nerve and spinal cord in patients with NMOSD occur frequently during the disease and may precede ON or TM by months or years (7–9). Thus, the international panel for NMO diagnosis has updated the definition of NMOSD to include the presence or absence of anti-AQP4 antibody (2). A diagnosis of NMOSD with anti-AQP4 antibody requires several core clinical characteristics, including clinical syndromes or magnetic resonance imaging (MRI) findings related to optic nerve, spinal cord, area postrema, other brainstem locations, diencephalic, or cerebral presentations (2). Recently, some cases have been reported with abnormalities in skeletal muscle and retinal cells expressing AQP4, characterized by the presence of immune complex deposition and AQP4 loss (10, 11). Therefore, manifestations in patients positive for AQP4 antibody are heterogeneous.

Surprisingly, despite the interest in brain abnormalities of patients with NMO/NMOSD, patients displaying pure brain symptoms have been rarely reported (7, 9, 12). Here, we describe the initial and follow-up clinical manifestations of patients with NMOSD who initially presented with different phenotypes, especially those with pure brain symptoms.

## Patients and Methods

### Patients

This retrospective study was approved by the Ethics Committee of the Second Affiliated Hospital of Guangzhou Medical University, China. All patients provided informed consent in the present study. Data analysis was performed based on the Chinese laws for data protection.

Consecutive patients positive for AQP4 antibodies, as detected retrospectively using a cell-based assay at the Second Affiliated Hospital of Guangzhou Medical University, were recruited until August 2015. The following data were acquired from each patient’s medical record: age, sex, medication, number of demyelinating events, clinical characteristics, and cerebral spinal fluid (CSF) protein levels and white cell counts. The Expanded Disability Status Scale (EDSS) (13) was conducted in these patients during follow-up at their most recent interview. Relapse was defined as objective worsening of new neurological symptoms that lasted at least 24 h and was preceded by disease stability for at least 1 month.

The patients were diagnosed as NMO/NMOSD based on the 2006 NMO diagnostic criteria (14) and the recent international panel guidelines (2). Cases of longitudinally extensive transverse myelitis (LETM) and acute partial transverse myelitis (APTM) were confirmed using MRI (15, 16). Cases of ON were defined by acute or subacute, unilateral or bilateral vision loss.

The recruited patients were divided into the following four groups based on initial disease manifestation: (i) with ON (NMOSD-ON^+^); (ii) with TM (NMOSD-TM^+^); (iii) without ON and TM (NMOSD-ON^−^TM^−^); and (iv) with simultaneous ON and TM NMO.

### AQP4 Antibody Testing

All CSF and serum samples were stored at −80°C. AQP4 antibodies were detected with a cell-based assay using a commercially available kit (Euroimmun, Luebeck, Germany) or by transfection of HEK293T cells with a construct containing human AQP4-M1 and AQP4-M23 genes.

### Statistical Analysis

All statistical analyses were conducted using Statistical Program for Social Sciences version 11.0 (SPSS, Chicago, IL, USA) software. The χ^2^-test was used for binary and categorical data. One-way ANOVA and Mann–Whitney *U* tests were used for continuous variables. A Kaplan–Meier analysis was performed to evaluate survival (time to relapse, conversion to NMO). The Kaplan–Meier analysis was compared between groups using log-rank tests. Values of *p* less than 0.05 were considered significant.

## Results

### Patient Demographics

A total of 292 patients with positive AQP4 antibodies were included in this retrospective study. This cohort comprised 253 females and 49 males (a female to male ratio of 6.49). Among these 292 participants, 178 (61%) were diagnosed with NMO and 114 (39%) with NMOSD based on their most recent follow-up (2) (Table 1). Their mean age at onset was 38.1 ± 14.5 years (range, 4–79 years); 22 of the 292 patients (7.53%) were older than 60 years at disease onset, and 10 (3.42%) were under 18 years old.

**Table 1:** **Final diagnosis and distribution of patients in three subgroups**.

Groups	*N* (%) of patients	Age at onset (median)	Female/male	No. (%) of immunosuppressive therapy^a^
(NMOSD)-ON^−^TM^−^	70 (100)	30	60/10	7 (10)
NMO*	38 (54.3)	30	35/3	3 (7.9)
RLETM	8 (11.4)	39	7/1	1 (12.5)
APTM	3 (4.3)	28	2/1	0
MON	3 (4.3)	30	3/0	0
RON	2 (2.9)	28.43	1/1	0
Others^b^	16 (22.9)	26	12/4	3 (18.8%)
NMOSD-ON^+^	95 (100)	37	83/12	22 (23.2%)
NMO*	78 (82.1)	38	69/9	20 (25.6%)
RON	12 (12.6)	37	9/3	2 (16.7%)
MON	5 (5.3)	25	5/0	0
NMOSD-TM^+^	116 (100)	40	98/18	10 (8.6)
NMO*	51 (44.00)	42	44/7	5 (9.8)
RLETM	40 (34.5)	40	34/6	5 (12.5)
MLETM	22 (19.0)	43	17/5	0
APTM	3 (2.6)	36	3/0	0 (0)
NMO	11 (100)	49	11/0	2 (6.8)

*NMOSD, neuromyelitis optica spectrum disorder; NMO, neuromyelitis optica; RLETM, recurrent longitudinal extensive transverse myelitis; MLETM, monophasic LETM; APTM, acute partial transverse myelitis; RON, recurrent optic neuritis; MON, monophasic ON*.

*^a^Long therapy with azathioprine, cyclophosphamide, methotrexate, and mycophenolate mofetil*.

*^b^Patients without optic neuritis (ON) and transverse myelitis (TM)*.

*Significantly different among the three groups (*p* < 0.0001).

**Figure 1:**
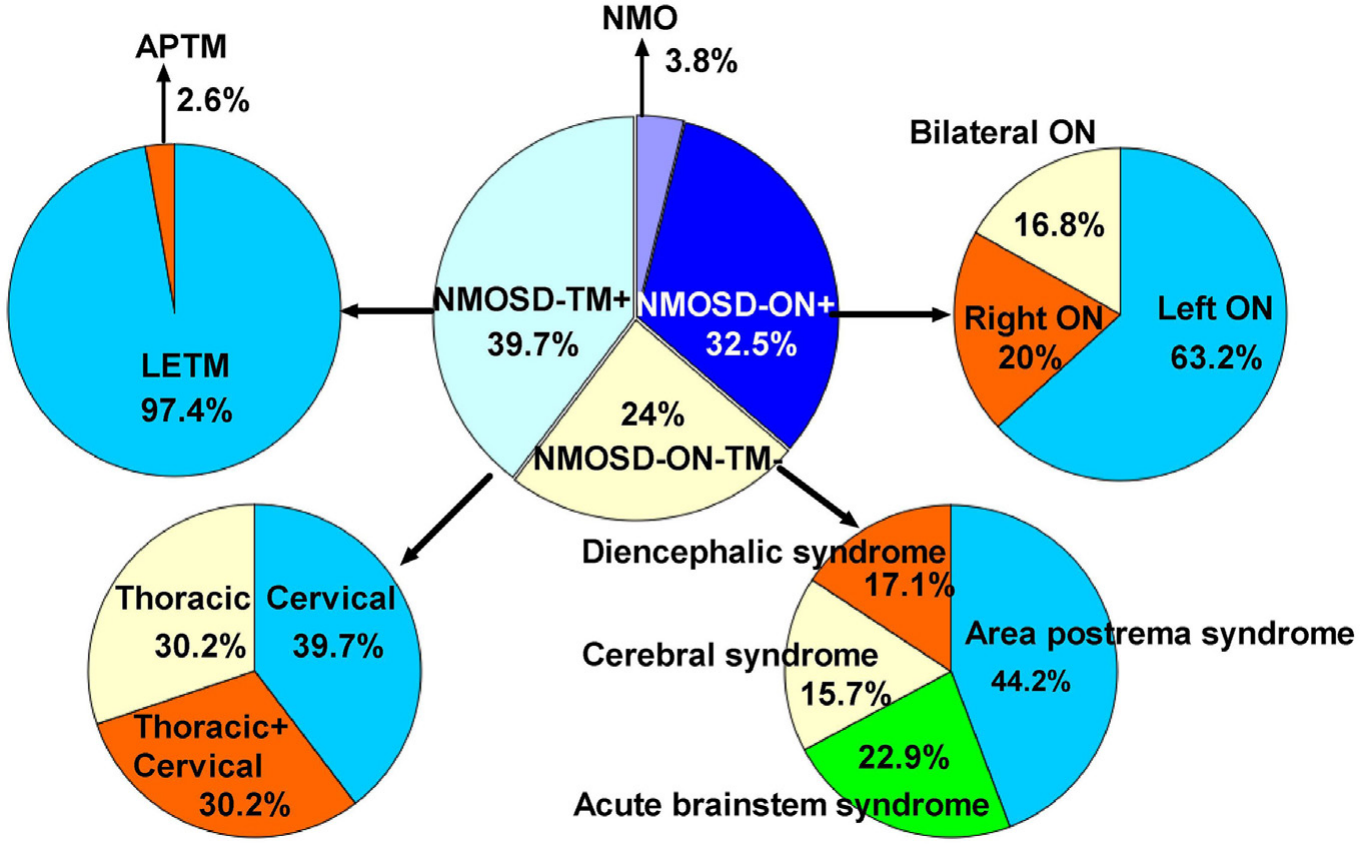
**Distribution of initial manifestations status in 292 patients**. NMOSD, neuromyelitis optica spectrum disorder; ON, optic neuritis; TM, transverse myelitis; APTM, acute partial transverse myelitis; LETM, longitudinally extensive transverse myelitis; NMOSD-ON−TM−, patient initial manifestation without ON and TM; NMOSD-ON+, patient initial manifestation with ON; NMOSD-TM+, patient initial manifestation with TM; neuromyelitis optica (NMO), patient initial manifestation with simultaneous ON and TM.

**Figure 2:**
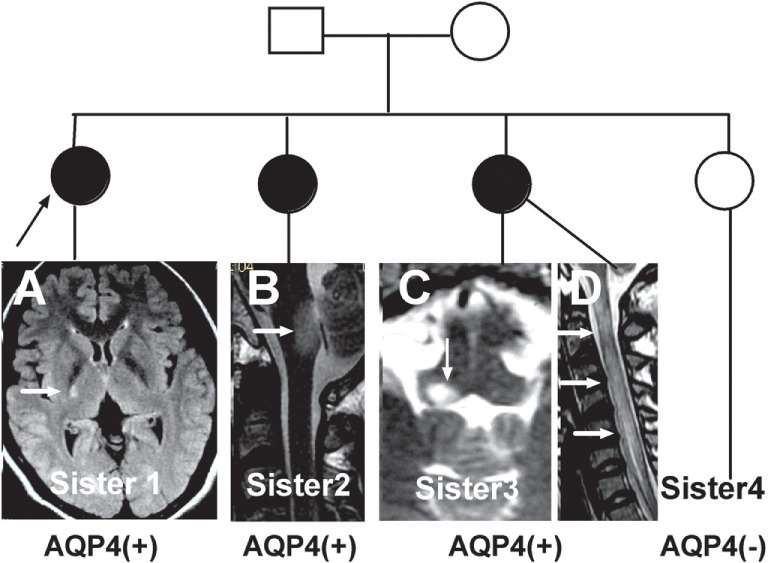
**Three neuromyelitis optica/neuromyelitis optica spectrum disorder cases from the same family**. **(A)** Case one is the oldest sister with autoimmune nephritis and the proband in this family. She experienced left limb weakness in 2004. A recent magnetic resonance imaging (MRI) scan showed a residual lesion in the posterior limb of internal capsule (arrow). **(B)** Case two is the second sister who experienced intractable hiccups and nausea and inappropriate antidiuretic hormone secretion in 2011. Her MRI showed a lesion in the dorsal medulla (arrow). **(C,D)** Case three, the third sister, had typical optic neuritis and transverse myelitis. Her MRI scan showed a lesion in the dorsal medulla and longitudinally extensive transverse myelitis (arrow).

The initial symptoms of the four groups are shown in Figure 1. Group (i) comprised 95 patients (32.5%, 95/292) diagnosed with ON (NMOSD-ON^+^) at onset. The disease started with isolated left ON in 60/95 cases (63.2%), isolated right ON in 19 (20%), and simultaneous bilateral ON in 16 (16.8%). Group (ii) consisted of 116 patients (39.7%, 116/292) diagnosed with TM (NMOSD-TM^+^) at onset. The disease started with LETM in 113/116 cases (97.4%) and APTM in 3/116 (2.6%) cases. Cervical lesions were found in 46/116 (39.7%), thoracic lesions in 35/116 (30.2%), and simultaneous cervical and thoracic lesions in 35/116 (30.2%) patients. Group (iii) was composed of 70 patients (24%, 70/292) without ON and TM (NMOSD-ON^−^TM^−^) at onset. The main brain symptoms observed included area postrema syndrome with hiccups or nausea and vomiting (44.2%, 31/70), acute brainstem syndrome (22.9%, 16/70), acute diencephalic clinical syndrome with NMOSD-typical diencephalic MRI lesions (17.1%, 12/70), and symptomatic cerebral syndrome (15.7%, 11/70). At the most recent interview, 14 patients (4.8%, 14/292) had confirmed episodes involving brain abnormalities only, without ON or TM, of which the duration was >12 months for 12 patients, 8 (57.1%) of whom experienced relapse. None of these patients were diagnosed as having NMO or NMOSD prior to the AQP4 antibody test. Two patients were from the same family, and their younger sister had been diagnosed with typical NMO with bilateral ON and LETM (Figure 2). Two patients had comorbidity of autoimmune nephritis. One case had comorbidity of anti-*N*-methyl-d-aspartate receptor encephalitis. Group 4 was composed of 11 patients (3.8%) diagnosed with simultaneous ON and TM (NMO) at onset.

### Comparison of NMOSD Phenotypes at Onset

The characteristics and phenotypes of the patients (*n* = 174) in the three NMOSD groups (NMOSD-ON^+^, NMOSD-TM^+^, and NMOSD-ON^−^TM^−^) who had complete MRI, CSF, demographic, and clinical data were compared. These three groups showed no significant differences in the sex ratio, disease duration, and number of relapsing cases (*p* > 0.05). However, the age at onset was lower in the NMOSD-ON^−^TM^−^ group than that in the other two groups (*p* < 0.005). The interval from the first episode to relapse (relapse-free time) was shorter in the NMOSD-ON^−^TM^−^ group than that in the NMOSD-TM^+^ group (*p* = 0.027). In addition, a significant difference was found among these groups for the NMO conversion (*p* < 0.0001). The CSF white cell count and protein level were significantly higher in the NMOSD-ON^−^TM^−^ group than those in the other two groups. The NMOSD-ON^−^TM^−^ group had lower EDSS scores than the other two groups in the most recent follow-up. Brain MRI abnormalities in area postrema and hemisphere lesions were more frequent in the NMOSD-ON^−^TM^−^ group (*p* < 0.005; Table 2), whereas brain MRI abnormalities were similar between the NMOSD-ON^+^ and NMOSD-TM^+^ groups. Pure cervical cord lesions were more frequent in the NMOSD-ON^−^TM^−^ group (*p* < 0.01).

**Table 2:** **Demographic and paraclinical characteristics in three subgroups with valid data**.

Characteristics	NMOSD-ON^−^TM^−^	NMOSD-TM^+^	NMOSD-ON^+^	p1	p2	p3

	(*n* = 53)	(*n* = 57)	(*n* = 64)			
Age onset (years)	31.6 ± 17.8	41.6 ± 14.2	37.0 ± 14.2	<0.0001	0.012	NS
Age onset < 30 years, *n* (%)	31 (58.5)	10 (17.5)	18 (28.1)	<0.0001	0.001	NS
Age onset >40 years, *n* (%)	16 (30.2)	30 (52.6)	25 (39.1)	0.017	NS	NS
Age onset >50 years, *n* (%)	5 (9.4)	16 (28.1)	13 (20.3)	0.013	NS	NS
Female/male	47/6	52/5	55/9	NS	NS	NS
Duration (months)	68.8 ± 58.8	76.6 ± 65.6	79.7 ± 68.4	NS	NS	NS
Relapsing cases, *n* (%)	48 (90.6)	48 (84.2)	62 (96.9)	NS	NS	NS
Relapse-free time (months)^a^	4 (1–96)	14 (2–312)	8 (1–120)	0.027	NS	NS
Neuromyelitis optica (NMO)-free time (months)^b^	24 (1–156)	24 (2–156)	16 (2–223)	NS	NS	NS
Meeting 2006 NMO criteria, *n* (%)	31 (57.4%)	21 (36.8%)	58 (90.6)	<0.0001	<0.0001	<0.0001
CSF protein (g/L)	0.44 ± 0.28	0.37 ± 0.20	0.27 ± 0.15	0.041	0.012	NS
CSF pleocytosis, *n* (%)	31 (58.5)	22 (38.6)	31 (48.4)	NS	NS	NS
CSF cells (no./mm^3^)	8 (0–325)	5 (0–161)	5 (0–98)	0.005	0.050	NS
Median EDSS (range)	3 (1–10)	5 (1–10)	5 (1–10)	0.008	0.010	NS
EDSS ≤ 3, *n* (%)	27 (50.9%)	10 (17.5)	16 (25)	<0.0001	<0.0001	<0.0001
EDSS ≥ 6, *n* (%)	10 (18.9%)	17 (29.8)	20 (31.3)	NS	NS	NS
Death, *n* (%)	2 (3.8)	2 (3.5)	2 (3.1)	NS	NS	NS
Brain NMO lesions in history						
Area postrema lesions, *n* (%)	32 (60.4)	11 (19.3)	14 (21.9)	<0.0001	<0.0001	NS
Brain stem lesions, *n* (%)	9 (17.0)	5 (8.8)	4 (6.3)	NS	NS	NS
Diencephalic lesion, *n* (%)	25 (47.2)	3 (5.3)	9 (14.1)	<0.0001	<0.0001	NS
Cerebral lesion, *n* (%)	15 (28.3)	2 (3.5)	4 (6.3)	0.001	0.001	NS
Spinal cord lesions in history	37 (69.8)	57 (100)	57 (89.1)	<0.0001	0.009	0.010
LETM, *n* (%)	30/37 (81.1)	55/57 (96.5)	47/57 (82.5)	0.013	NS	0.015
Cervical lesions, *n* (%)	24/37 (64.9)	21/57 (36.8)	20/57 (35.1)	0.008	0.005	NS
Thoracic lesions, *n* (%)	6/37 (16.2)	16/57 (28.1)	25/57 (43.9)	NS	0.005	NS
Cervical + thoracic lesions, *n* (%)	7/37 (18.9)	20/57 (35.1)	12/57 (21.1)	NS	NS	NS

*NMOSD, neuromyelitis optica spectrum disease; LETM, longitudinal extensive transverse myelitis; EDSS, Expanded Disability Status Scale; NMOSD-ON^+^, patient initial manifestation with ON; NMOSD-TM^+^, patient initial manifestation with TM; NMOSD-ON^+^TM^+^, patient initial manifestation with simultaneous optic neuritis (ON) and transverse myelitis (TM); CSF, cerebral spinal fluid; p1, comparison between NMOSD-ON^−^TM^−^ and NMOSD-TM^+^ patients; p2, comparison between NMOSD-ON^−^TM^−^ and NMOSD-ON^+^ patients; p3, comparison between NMOSD-ON^+^ and NMOSD-TM^+^ patients*.

*^a^Duration from the first attack to the first relapse*.

*^b^Duration from the first attack to diagnosis of NMO*.

### Follow-up and Kaplan–Meier Analysis

Patients were included in this analysis if they had validated relapsing events that occurred from the time of the initial incident to the most recent interview and the duration of those symptoms was greater than 12 months. Thus, follow-up data were analyzed for 226 patients in the three NMOSD groups (NMOSD-ON^+^, NMOSD-TM^+^, and NMOSD-ON^−^TM^−^).

Among the 61 patients analyzed in the NMOSD-ON^−^TM^−^ group, 60 (98.4%) experienced relapse, and 31 patients (50.8%) met the NMO diagnostic criteria during follow-up (12–268 months). Among the 80 patients analyzed in the NMOSD-TM^+^ group, 72 patients (90%) experienced relapse, and 34 (42.5%) were diagnosed with NMO during follow-up (12–324 months). Among the 85 patients analyzed in the NMOSD-ON^+^ group, 80 patients (94.1%) experienced relapse, and 71 (83.5%) met the NMO diagnostic criteria during follow-up (12–346 months). The conversion to NMO in the NMOSD-ON^+^ group was greater than that in the other tow groups (*p* < 0.0001).

**Figure 3:**
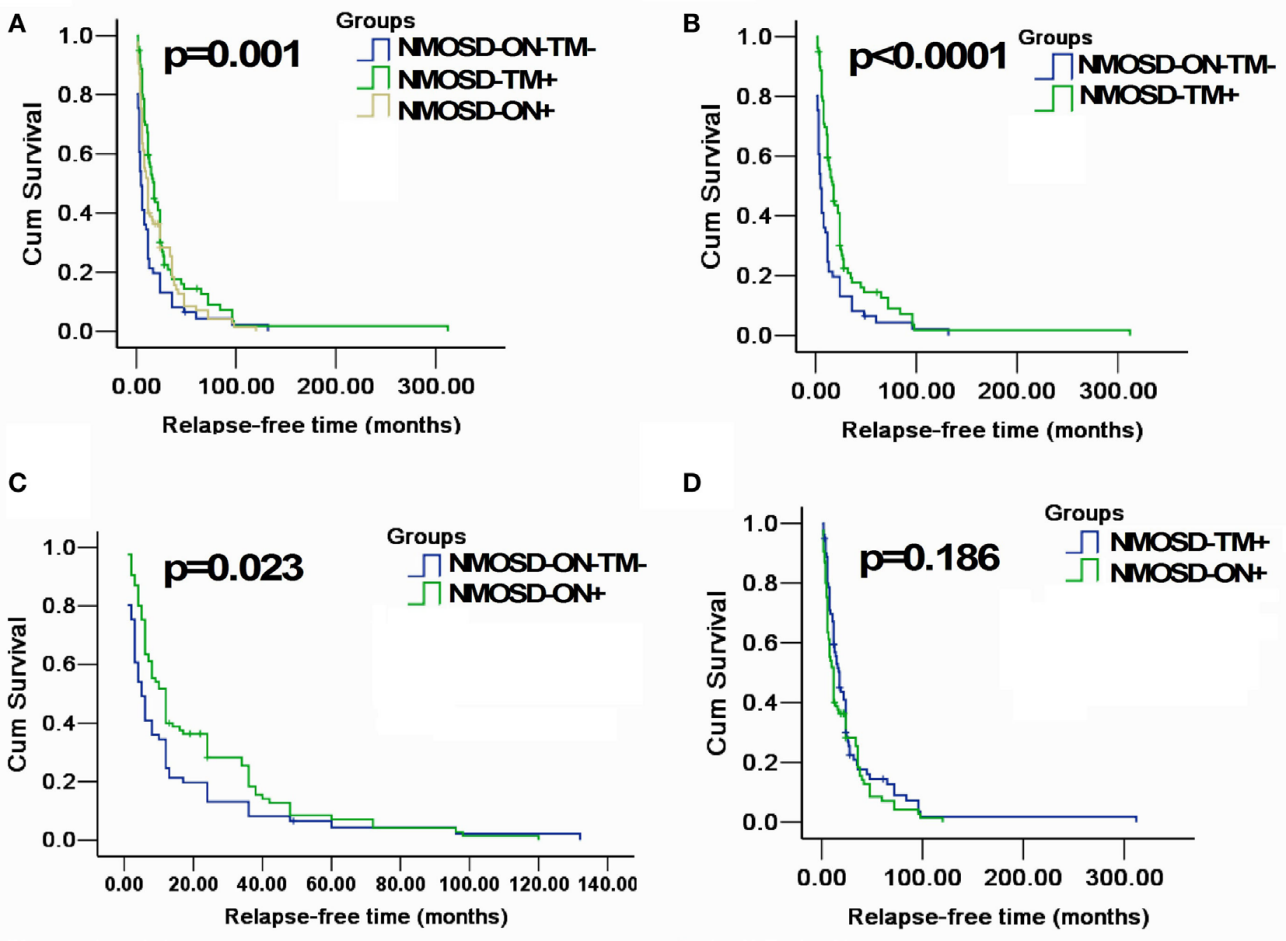
**Kaplan–Meier analyses stratified by different groups: considering the end point is the first relapse**. **(A)** Kaplan–Meier analysis revealed that patients in three groups would experience different relapsing time after the first attack (*p* = 0.001); **(B,C)** Kaplan–Meier analysis revealed that patients in MOSD-ON^−^TM^−^ group would experience earlier relapse after the first attack and was significantly different neuromyelitis optica spectrum disorder (NMOSD)-TM^+^ group and NMOSD-ON^+^ (*p* < 0.05); and **(D)** Kaplan–Meier analysis revealed no significant differences in time to the first relapse between NMOSD-ON^+^ and NMOSD-TM^+^ group (*p* = 0.186).

**Figure 4:**
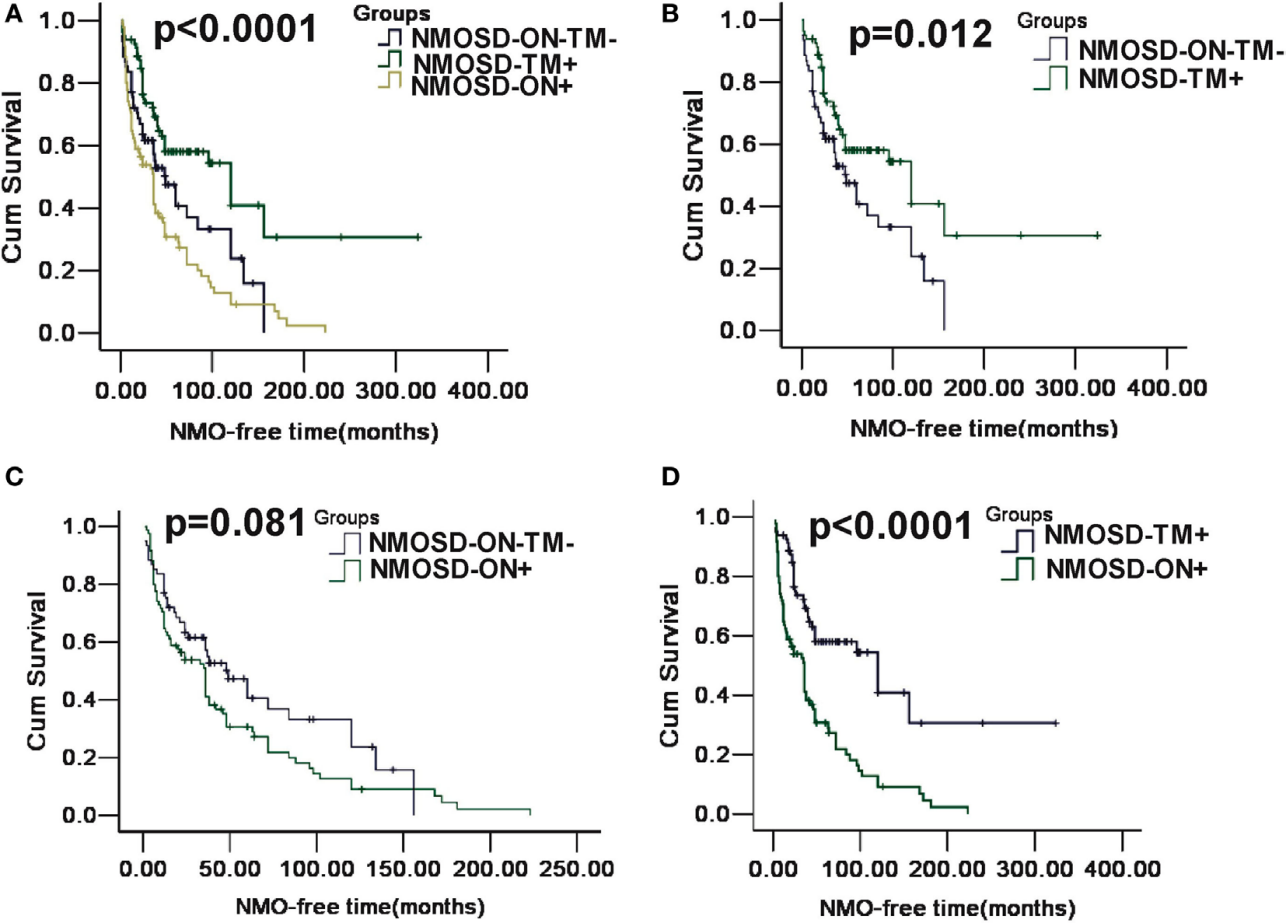
**Kaplan–Meier analyses stratified by different groups: considering the conversion to neuromyelitis optica (NMO)**. **(A)** Kaplan–Meier analysis revealed that patients in three groups would experience different time of NMO conversion after the first attack (*p* < 0.0001); **(B)** Kaplan–Meier analysis revealed that patients in MOSD-ON^−^TM^−^ group would experience earlier NMO event after the first attack and was significantly different neuromyelitis optica spectrum disorder (NMOSD)-TM^+^ group (*p* = 0.012); **(C)** Kaplan–Meier analysis revealed no significant differences in time to the NMO event between NMOSD-ON^+^ and MOSD-ON^−^TM^−^ group (*p* = 0.081); and **(D)** Kaplan–Meier analysis revealed patients in NMOSD-ON^+^ group would experienced earlier NMO event after the first attack and was significantly different NMOSD-TM^+^ group (*p* < 0.0001).

A Kaplan–Meier analysis revealed that compared with NMOSD-TM^+^ cases, NMOSD-ON^−^TM^−^ patients experienced significantly earlier relapses after the first attack (*p* = 0.002). However, these two groups showed a similar relapse rate at late follow-up (>100 months) (Figure 3). Furthermore, compared with NMOSD-ON^+^ cases, NMOSD-ON^−^TM^−^ cases also experienced significantly earlier relapses (*p* = 0.023), although both of these groups had similar relapse rates at >50 months. The Kaplan–Meier analysis also revealed that the interval from first attack to NMO conversion differed among the groups (Figure 4). The median time of 120 months [95% confidence interval (CI): 40.6–199.4 months] in NMOSD-TM^+^ cases was significantly longer than that of 49.0 months for the NMOSD-ON^−^TM^−^ cases (95% CI: 26.8–71.2 months, *p* = 0.012) and 36.0 months for the NMOSD-ON^+^ cases (95% CI: 25.3–46.7 months, *p* < 0.0001).

## Discussion

The present study found that initial manifestations without TM or ON were not uncommon in NMOSD patients. In all the patients examined, the onset of brain/brainstem lesions was more frequent than that previously shown in a large study (12). We observed that 19.5% (57/292) of patients had involvement in area postrema/brainstem, including displaying intractable hiccups and nausea (IHN) (10.6%, 31/29). Although increasing numbers of NMOSD patients with brain/brainstem-onset manifestations have been reported, such patients without ON and TM in NMOSD have been rarely reported in previously conducted large studies. For example, a relatively large study (12) reported 18% of patients positive for AQP4 antibodies without TM and ON presented with brain symptoms as their initial manifestation. In Japan, 28.6% (10/35) of cases showed IHN preceding ON and TM, and IHN was preceded by an episode of viral infection (17). Apiwattanakul et al. reported that the initial presenting symptom of NMO was intractable vomiting in 12% of AQP4 antibody-positive patients (8). In addition, hiccup and nausea often preceded neurological symptoms such as ON and TM, and 14% (10/70) of newly identified AQP4-IgG-positive patients had nausea and vomiting as the initial presenting symptoms of NMOSD (18). Most brainstem attacks were first events and were regarded as monophasic brainstem symptoms until follow-up (19, 20). However, a large study of 106 NMOSD patients seropositive for AQP4 antibodies reported that 4.7% (5/106) of patients had initial brain/brainstem manifestations without TM and ON (21). Another study from multiple centers showed only 2.3% (4/175) of NMOSD patients had brainstem-onset involvement (22). Our study found that 10.6% of patients had IHN, which is comparable to that found in a recent large study (8, 18). Therefore, IHN may have been underestimated in some previous studies because acute clinical events without TM or ON may have been overlooked, and some patients may have experienced lesion resolution. Although these patients had brain disease involvement before an ON or TM episode, most brain attacks were first events and almost all developed into NMO/NMOSD with ON and/or TM at follow-up (19, 20).

All groups in our cohort consisted of more females than males, consistent with observations in previous studies. Furthermore, the mean age at disease onset in patients presenting with ON was significantly lower than that in patients presenting with TM (9, 14, 21, 22). In our study, patients with disease onset were >40 years of age; however, similar numbers presented with TM (52.6%) or ON (39.1%) (*p* > 0.005), indicating a predominance of disease onset in young patients without TM and ON. Their mean age at disease onset was similar to that reported in previous studies showing a younger mean age at disease onset in patients with only brain/brainstem manifestations (9, 12). Thus, AQP4-mediated brain/brainstem disease may occur in patients younger than those with ON and TM, supporting age-dependent anatomical susceptibility differences or differences in AQP4 antibody accessibility of the target organs (23). However, Afro-Caribbean patients (9) reportedly have a younger age of disease onset, indicating ethnicity may be an important factor. Studies in China examining such differences would also be warranted.

The Kaplan–Meier analysis of our cohort demonstrated that >50% of patients experienced a relapse within 1.5 years of disease onset, and almost all experienced relapse within 10 years (Figure 3). Patients presenting with only brain/brainstem lesions had a shorter relapse-free time than those with ON or TM, a result similar to previous findings (22). There was a trend toward ON-onset patients relapsing sooner than TM-onset patients, which is in contrast to other studies in which AQP4 antibody-positive patients with TM-onset had earlier relapse than ON-onset patients (21, 22). Patients without ON or TM at onset had more lesions in areas without an intact blood–brain barrier, indicating that AQP4-mediated relapsing episodes may precede other neurological symptoms. However, it is unclear why lesions in these areas are less common than ON and spinal cord lesions (17).

We observed a different median time for NMO to develop in the three subgroups, as reported previously (21); however, the median time was relatively short in our cohort. Additionally, patients presenting with ON had a higher probability of developing NMO over time than those presenting with TM or brain/brainstem episodes. This may be because AQP4-mediated monophasic or relapsing ON is underrecognized, and prophylactic immunosuppressant therapy is not readily available (21). However, in our cohort, >60% of patients with TM did not develop NMO based on the 2006 diagnostic criteria (14), and even relapsing patients experienced a delay of >20 years. Therefore, our results support the ideas that AQP4-mediated disease is not synonymous with the classical description of NMO and that the first manifestations reflect different NMO phenotypes.

In our cohort, fewer patients presenting with brain disease progressed to NMO (31/61, 50.8%) compared with those in a study examining Korean patients (10/15, 66.7%) (12), which may be associated with the small sample size used in their study. Interestingly, some of our patients lacked typical ON and TM at onset attack, with an interval of years between onset attack and first episodes of ON or TM. However, AQP4 antibody-positive patients with manifestations suggesting long-term brain/brainstem involvement without ON and TM, especially those with a relapsing course, have been rarely reported (7, 9, 24–27). We found 12 patients (4.1%, 12/292) with episodes involving the brain without ON or TM over years, which is higher than the 2.4% (7/289) detected in Japan (9). In the present retrospective analysis, all patients with manifestations suggestive of brain involvement at onset were misdiagnosed with other diseases because of their atypical and complicated manifestations. Isolated lesions in the supratentorial region of the brain were rarely observed in the present study and have only been described in single case reports (7, 24–27). Atypical manifestations make diseases more difficult to diagnose, indicating AQP4 antibody-positive patients with long-term relapsing symptoms other than ON or TM may be easily misdiagnosed without the detection of AQP4 antibodies. Additionally, some patients with phenotypic presentations suggestive of brain disease presented concomitantly with an immune disorder involving another organ; for example, two patients had immunological disorders of the kidney. Previously, recurrent hyperCKemia accompanying AQP4-IgG seropositivity reflected pathogenic IgG targeting of skeletal muscle AQP4 (10). However, no typical AQP4 loss in kidney could be found with biopsy in our cases, although the AQP4 expression was relatively weaker than that in the control (not shown). Therefore, whether autoimmune AQP4 in the kidney is associated with NMOSD should be examined further, because AQP4-IgG seropositive cases do not meet the present definition of NMOSD, indicating that they may be autoimmune AQP4 channelopathies (3).

Mortality was significantly different in the three subgroups. First, the most recent median EDSS scores were lower in patients with brain/brainstem manifestations compared with those with ON or TM attacks at onset. Second, further analysis showed that compared with patients with ON, more patients with brain/brainstem manifestations had EDSS scores <3.0, and there was a trend toward fewer brain/brainstem-onset patients having EDSS scores >6.0. The prognosis for ON-onset patients was worse, and this may be related to a high proportion of these patients with NMO development, resulting in visual and motor disabilities. Although a low proportion of TM-onset patients developed NMO, their older age and increased proportion with LETM may be important factors for mortality. Older-onset patients are reportedly more likely to present with LETM and have a high risk of developing motor disability (21). It was previously shown that brain lesions with AQP4 autoimmunity in patients with NMOSD are accompanied by discontinued vasogenic edema (14). Thus, brain/brainstem-onset patients with recurrent brain/brainstem lesions may be predisposed to revisable edema without axonal injury resulting in slight persistent disability. Effective immunosuppressive treatment with good tolerance may prevent relapse or conversion to NMO. However, although some patients followed immunosuppressive regimes, >80% of these patients started treatment with only high-dose corticosteroids, tapering over several months to low-dosage steroids. The potential benefit of immunosuppressive therapy was not observed in the present cohort because of low use. In China, long-term immunosuppressive treatment is limited by adverse effects, patient compliance, and poor doctor–patient relationships (28). We believe that using recommended first-line long-term immunosuppression would provide a better prognosis.

The present study had some limitations. First, there was a selection bias because of the retrospective nature of the study and because some patients had incomplete data. Furthermore, a recall bias might result from data collection acquired from a patient’s medical record or interview. Second, detection of AQP4 was retrospective; therefore, AQP4 status at the initial episode was unknown. Although prospective studies are important, the current study is valid and will help clinicians treat different phenotypes of NMOSD. Third, our study focus is limited on the AQP4-positive NMOSD patients, which allows for a clear disease population, but leaves out AQP4-negative NMOSD. In particular in the latter disease group, would an analysis of the different phenotypes be of diagnostic importance and guidance for treatment decision. Given the recent insights into the role of antibodies against native conformational myelin oligodendrocyte glycoprotein (MOG) (29), embedding the data on NMOSD with mere brain involvement in this context would give a more comprehensive and up-to date picture of the patients with an “NMOSD phenotype” and brain involvement. Therefore, detection of MOG antibody is necessary in our further study.

In summary, we observed significant differences in clinical features during follow-up among NMOSD patients who presented with different initial manifestations. Patients who presented without ON and TM as their initial manifestations were younger at onset and had earlier relapses and more brain abnormalities but better prognosis than those who presented with ON and TM. The conversion to NMO in patients with TM at onset was lower than that in the other patient groups. Furthermore, the conversion to NMO was more frequent in patients with ON at onset than that in patients with brain/brainstem manifestations at onset. Patients with long-term AQP4 autoimmunity in the brain in the absence of ON or TM were not common.

## Author Contributions

Study concept and design: YL, XC, and CG. Acquisition of data: YL, JL, LW, SL, XC, WQ, YY, XZ, NY, MG, YC, ZW, and QS. Analysis and interpretation of data: YL and CG. Drafting of the manuscript: YL, LW, and JL. Critical revision of the manuscript for important intellectual content: YL, JL, LW, Zhong, and CG. Obtained funding: YL and CG. Administrative, technical, and material support: YL, JL, LW, SL, XC, WQ, YY, XZ, NY, MG, and YC.

## Conflict of Interest Statement

The authors declare that the research was conducted in the absence of any commercial or financial relationships that could be construed as a potential conflict of interest.
